# Using an Electronic Decision Support Tool to Reduce Inappropriate Polypharmacy and Optimize Medicines: Rationale and Methods

**DOI:** 10.2196/resprot.5543

**Published:** 2016-06-10

**Authors:** Amber Young, June Tordoff, Susan Dovey, David Reith, Hywel Lloyd, Murray Tilyard, Alesha Smith

**Affiliations:** ^1^ New Zealand’s National School of Pharmacy University of Otago Dunedin New Zealand; ^2^ Department of General Practice and Rural Health Dunedin School of Medicine University of Otago Dunedin New Zealand; ^3^ Dunedin School of Medicine University of Otago Dunedin New Zealand; ^4^ Best Practice Advocacy Centre (BPAC) Dunedin New Zealand

**Keywords:** polypharmacy, decision support systems, clinical, drug-related side effects and adverse reactions, drug interactions, primary health care, inappropriate prescribing, medication therapy management

## Abstract

**Background:**

Polypharmacy and inappropriate continuation of medicines can lead to a significant risk of adverse drug events and drug interactions with patient harm and escalating health care costs as a result. Thorough review of patients’ medications focusing on the need for each drug can reduce the potential for harm. Limitations in performing effective medicine reviews in practice include consultation time constraints and funding for pharmacy services. We will aim to overcome these problems by designing an automatic electronic decision support tool (the medicines optimization/review and evaluation (MORE) module) that is embedded in general practice electronic records systems. The tool will focus on medicines optimization and reducing polypharmacy to aid prescribers in reviewing medicines and improve patient outcomes.

**Objective:**

The objectives of this study are: (1) to develop an electronic decision support tool to assist prescribers in performing clinical medication reviews with a particular focus on patients experiencing multimorbidity and polypharmacy, and (2) evaluate and assess the use of the electronic decision support tool, providing pilot data on its usefulness in supporting prescribers during consultations with patients.

**Methods:**

The first three study phases involve development of clinical rules outlining clinical interventions and the creation and validation of the MORE decision support tool. Phase four is a community-based, single-blind, prospective, 6-month controlled trial involving two interventions and two control general practices, matched for practice demographics. We will be measuring the number of times prescribers engage with the tool, total number of interventions suggested by the tool, and total number of times prescribers change medicines in response to recommendations. There will also be prospective follow-up of patients in the intervention group to examine whether changes to medications are upheld, and to determine the number of hospitalizations or emergency department visits within 6 months of a medicine intervention. Comparisons between control and intervention practices will measure the changes in proportions of patients with polypharmacy and inappropriately prescribed medicines before and after the introduction of the electronic decision support tool, proportions of patients receiving appropriate treatment in each practice, and changed, maintained, or improved health status, hospitalizations, and deaths in the study year. Initiation rates of inappropriately prescribed medicines will be measured as a secondary outcome. As well as external assessment of the extent of use and application of the tool, prescribers will receive monthly practice progress reports detailing the proportion of their patients experiencing polypharmacy and taking inappropriately prescribed medicines identified for review.

**Results:**

Phase one has now been completed and the decision support tool is under development. Final data analysis is expected to be available in December 2016.

**Conclusions:**

This study will establish whether the MORE decision support tool stands up to real world conditions and promotes changes in prescribing practice.

## Introduction

### Background

As the New Zealand population is rapidly ageing, there are increasing numbers of patients with more long-term conditions and taking more medicines. With declining organ function and introduction of comorbidities, increasing age may also influence the suitability of a patient’s long-term medications. Periodic assessment of patients’ medications should be undertaken, keeping in mind their remaining life expectancy, time until benefit of treatment, treatment targets, and goals of care [1].

Polypharmacy can be defined as the concurrent use of five or more medicines, and excessive polypharmacy, the use of 10 or more concurrent medicines [2]. Inappropriate polypharmacy occurs when more medicines are prescribed than are clinically indicated or when medicines are inappropriately continued [3]. Increasing the number of prescribed medicines greatly increases the risk of drug interactions and adverse drug events, resulting in iatrogenic patient morbidity [3-5]. The medical mantra of ‘First do no harm’ is at risk when patients’ are in danger of multimorbidity from cumulative prescribing of inappropriate medicines, particularly when this is compounded with altered pharmacodynamics from declining renal and hepatic function with age. Overall, there is a danger that patients’ medication regimens may begin to pose more risks than benefits to their health [5]. Between 2013 and 2014, 8.5% of the New Zealand population received five or more medicines and 2.6% received 11 or more medicines [2]. These proportions of the population with polypharmacy and hyperpolypharmacy are increasing in all age groups every year [2], particularly for 40- to 60-year olds [6].

Thorough review of patients’ medications focusing on the need for each drug can reduce the potential for harm [5,7]. A 2014 Cochrane review found that interventions to improve appropriate polypharmacy are beneficial in reducing inappropriate prescribing [3]. Despite the evidence, most people taking multiple medicines do not receive an annual comprehensive medicines review due to general practitioners’ limited consultation times. Tools exist to assist a review but are infrequently used due to being complex and time-consuming [8,9]. A scheme for collaborative medications reviews involving pharmacists exists in New Zealand (Medicines Therapy Assessment) but is funded in few regions. This deficiency is similar worldwide as many international models of primary care do not promote intensive medication reviews by a clinical pharmacist.

This study will attempt to overcome the problems in completing medication reviews by designing an automatic electronic decision support tool. These tools have been shown to influence prescriber performance, improve quality of care and patient outcomes [10], and reduce inappropriate prescribing [3]. The medicines optimization/review and evaluation (MORE) decision support tool will focus on medicines optimization to aid prescribers in reviewing medicines and improve patient outcomes while reducing inappropriate polypharmacy. The electronic decision support software will also provide continuous and reproducible medication reviews [11].

Recent studies have demonstrated benefits of electronic decision support tools, but the technology itself can become a burden on physicians’ time and patient management [12-15]. For successful implementation, decision support tools must be fast, reliable, and able to integrate into existing systems used in practice.

The 2012 review by Clyne et al [12] demonstrated that clinical decision support has potential to improve safe and effective prescribing in many different health care settings. Likewise, the review by Topinkova et al [16] from the same year found that decision support reduces prescribing errors. However, they also concluded that the real effect of these systems requires further study, focusing on health outcomes such as overall health care cost and patient morbidity and mortality. Further research is required to evaluate the acceptability of alerts to prescribers’ [16]. The 2014 Cochrane review, examined studies using specific validated screening tools or instruments, but did not include research on how doctors interact with the specific interventions, and based their recommendations for implementation of change on the recommendations presented to them [3].

This study will use an electronic decision support platform currently available in over 80% of New Zealand general practices and compatible with the most common patient management system in use. We expect that prescribers’ familiarity with the decision support platform will enable immediate uptake and application of the MORE tool. We will also investigate the interaction of the doctor with the decision support tool to evaluate its’ effectiveness and usefulness in practice.

In summary, the significance of this study is the development of a new decision support tool that uses each patient’s medical information to automatically undertake a medicines review and assist prescribers with patient management. Personalized medicine management strategies help ensure that patients receive the most appropriate care and avoids the risk of ‘alert fatigue’. Broad use of our tool is intended to improve clinical outcomes, and reduce health care use and cost. The evaluation and review of users’ feedback will enable the production of a practical and efficient decision support tool.

### Research Aims

The purpose of this study is to develop and test an electronic decision support tool (the MORE module) designed to assist prescribers in performing clinical medication reviews for patients experiencing multimorbidity and polypharmacy. The study will (1) provide pilot data on its usefulness in supporting prescribers during consultations with patients, and (2) test the hypothesis that there is no difference in the identification and management of patients with multiple medicines between the practices implementing the MORE decision support tool and usual care practices.

## Methods

### Phase One: The Development of the Clinical Rules for the MORE Decision Support Tool

**Figure 1:**
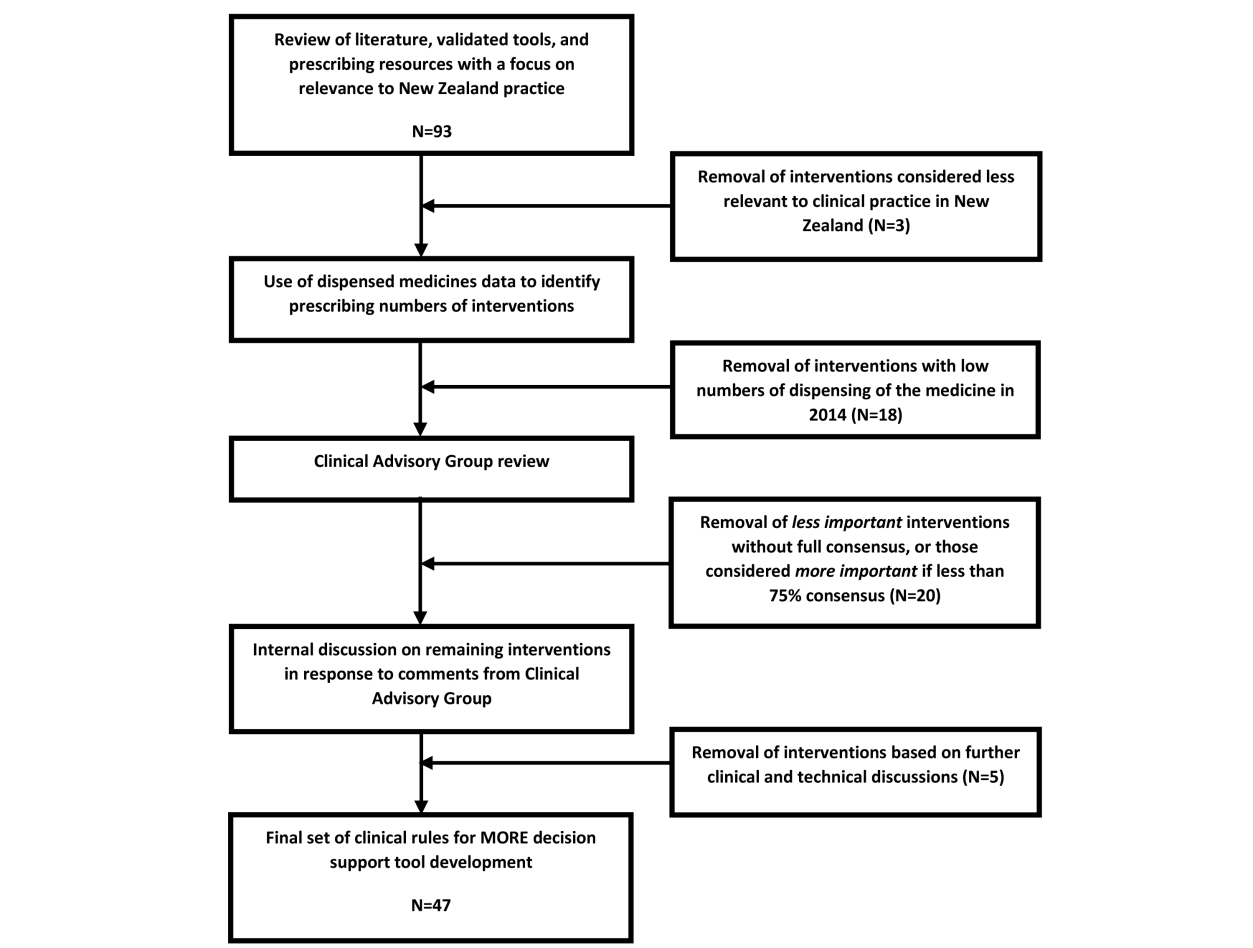
Development of the clinical rules for the MORE decision support tool.

Phase one involved the development of the intervention components to be incorporated into the MORE decision support tool. Literature reviews, internationally validated tools, and prescribing resources were used to identify possible intervention components (see Figure 1). A clinical advisory group was set up to provide expert opinion and direction for the development of the intervention components. This group consisted of 10 clinicians.

The intervention components focused on specific areas for change, including:

1. Reducing polypharmacy by targeting medicines with limited effectiveness, such as long-term use of benzodiazepines for insomnia, or antipsychotics in dementia

2. Stopping duplicate medication classes, such as duplicate antidepressant therapy

3. Reviewing doses and monitoring certain medications such as proton pump inhibitors, anticoagulants, and analgesia

To prioritize the list of interventions, medicines dispensing data were reviewed using the Pharmaceutical Collection, a national database of all community pharmacy dispensed medicines. Possible interventions for medicines not dispensed in large numbers (<5000 dispensing’s a year nationally) were excluded. Possible interventions remaining were reviewed by the clinical advisory group to determine the appropriateness of the recommendations for the New Zealand context. The group assessed the importance of each medicine intervention using a list of references provided and their own clinical expertise. International specialist opinion was also sought. Descriptions of the interventions were modified following comments by the group, and the proposed medicine interventions with the greatest consensus of agreement were selected as the final intervention components.

These final medicine intervention components are expected to have the most impact on patient care and polypharmacy, and were used to form the clinical rules of the MORE decision support tool. See Figure 1 for an outline of the intervention development process.

### Phase Two: Development of the MORE Decision Support Tool

Decision support programmers (from BPAC Inc) will use the medicine intervention components generated in phase one to create the clinical rules in the MORE decision support tool. This tool will automatically interact with the prescribing component of general practice electronic records systems and access individual patients’ demographic and clinical information to make recommendations to prescribers. The program developers have previously built the Best Practice Advocacy Centre (BPAC) decision support tools, ensuring that the look, feel, and function of the MORE tool are similar to that of the current BPAC decision support tools that are in widespread use in New Zealand. These tools are also currently in use in Australia and soon to be introduced into the United Kingdom.

**Figure 2:**
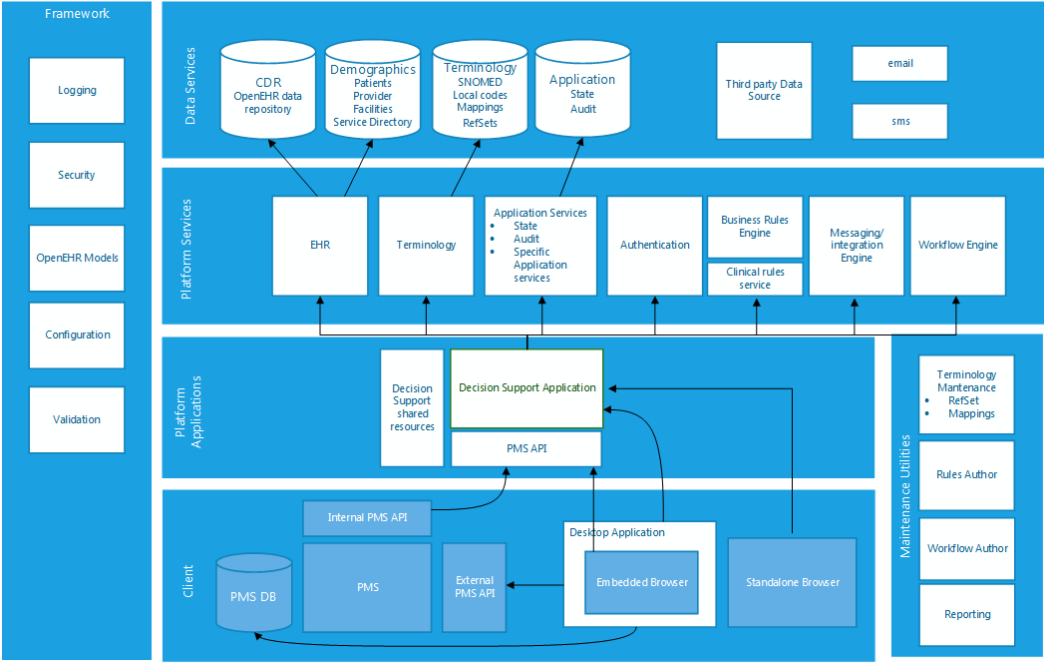
Platform architecture of the decision support tool.

The decision support architecture will be using open electronic health record (open EHR) for the patient object model, which is an open international standard. This allows for the inclusion of any coding system from any underlying patient management system (PMS) to be integrated into the patient object model. The patient object model also allows for integration with any PMS application program interface (API), allowing for rapid international roll out. Interoperability is also enabled through the mapping of drug codes, medical classification, laboratory codes, and measurement parameters to the systematized nomenclature of medicine clinical terminology (SNOMED CT) using an in-house ontology service. Furthermore, the clinical rules driving the prescribing advice reside in a rules engine, allowing additional rules and functionality to be added independently of any programming interfaces. Figure 2 shows the platform architecture of the decision support module. Not all components will be used for this study, such as the SMS text messaging (short message service, SMS) function, but will be available for future roll out. This platform architecture enables rapid scalability.

The MORE decision support tool will alert prescribers through the patient prompt: this analyses patient records at the time of consultation, notifying clinicians of any areas where action may be required. Recommendations will be based on individual patients’ data and therefore are specific for each patient.

Simple representation of targeted advice and prescribing alternatives is more effective than highlighting a medicine that may be inappropriate [17]. Therefore, this decision support tool will alert prescribers to medicines within patient records that are potentially inappropriate with suggested action based on each patient’s individual data. There will also be a link to electronic information supporting recommended actions, where relevant. The decision support tool will also allow linking to patient information and advice that can be printed.

### Phase Three: Validation of the MORE Decision Support Tool

The MORE decision support tool will be validated by challenging it with data from a de-identified historical database. This will ensure that the clinical rule set is working correctly. The decision support tool will be applied to a variety of patients in the database (patients with ≥10 medicines and patients with only a few medicines) to confirm that patient data are autopopulated into the decision support tool correctly and whether an alert is triggered by the appropriate information. This phase will also allow technical checks such as the linking to external information and printing functionality. Personal practitioner progress reports will also be generated to ensure this functionality works correctly.

### Phase Four: Application of the MORE Decision Support Tool

Phase four is the feasibility study of the MORE decision support tool using MORE care versus usual care to reduce unnecessary or inappropriate prescribing. The MORE decision support tool will be piloted for 6 months in two intervention general practices. A comparison in prescribing behavior and outcomes will be made between the practices and two matched control practices. This will establish whether the decision support tool stands up to real world conditions and causes a change in prescribing practices.

During the 6 months that the MORE decision support tool is active, prescribers will receive individual monthly progress reports that have been extracted remotely by BPAC analysts using the MORE decision support tool. The reports will detail the proportion of patients at their practice experiencing polypharmacy, and patients taking prescribed medicines identified for review.

This information will include patient demographics and the total number of times prescribers in the practice have engaged with the MORE decision support tool with resulting changes they may have implemented. They will also be informed of the clinical rules that, following the alert, have resulted in changes to patients’ therapy the most and the least number of times.

As the study progresses the reports will depict the changes in these variables over time. Using the decision support tool query builder, prescribers will also be able to access lists of patients experiencing polypharmacy and those taking prescribed medicines identified for review, any time during the project.

#### Study Setting

The MORE decision support tool will be provided to two intervention general practices recruited by invitation. In order for a practice to be considered for inclusion, it must be using the MedTech software, which is in use in over 80% of New Zealand practices. Practices will be excluded from the study if they are specialist practices (eg, a sexual health clinic, student health clinic), or if they have less than 1500 registered patients. Of the practices wishing to participate, two will be randomly assigned as the MORE decision support practices and two control practices will be matched according to practice patient demographics (mean age, gender, ethnicity, and geography (urban/rural)).

The MORE decision support tool will be added to the intervention and control practices’ suite of BPAC decision support tools, although in the control practices, the alerts will not be revealed at point-of-care. Instead, the type and frequency of the alerts that would have been shown will be recorded for comparison against those in the intervention group. The decision support tool can be added to the decision support platform remotely because it is a Web-based system that allows upgrades or revisions to existing decision support tools to be implemented through one centralized server. This will minimize disruption to the daily activities of study general practices.

#### Sample Size

Between 2013 and 2014, 11% of the New Zealand population used five or more medicines [2]. A sample size of 367 patients for each arm (the intervention and the control) will have 80% power to detect a difference in patients with polypharmacy of 20% at the 5% level of significance.

We intend to recruit four practices with 1500 or more registered patients. The average number of patients per practice in New Zealand is approximately 4000 [18], so our goal of practices with 1500 or more registered patients is achievable. Ethical approval has been obtained from the Health and Disability ethics committee (16/STH/7).

### Measures

This study will test the hypothesis that there is no difference between the practices using the MORE decision support tool and usual care practices in the identification of patients with polypharmacy and the identification and review of patients who have inappropriately prescribed medicines.

Due to the nature of the study, it is not possible to blind the general practices to the intervention. However, the data retrieval from the general practices is through an automated Web-based system and a blinded analysis of the measures comparing intervention and control groups will be undertaken. This allows for unbiased assessment of these outcomes.

We plan to assess the use of the MORE decision support tool by seeing how often general practitioners use the tool and follow its recommendations. We will calculate how often the alert for review was raised and compare this with changes in prescribing. The number of patients experiencing inappropriate medicines (as defined by the clinical rules in the decision support tool) and polypharmacy before and after the study will also be reviewed, as a proxy measure of effectiveness of the intervention. See Table 1 for detailed descriptions of study measures and timeframes.

**Table 1:** Study Outcomes and How They Will be Measured.

Outcomes	How this will be measured	Timeframe will this be measured
**Practitioners use the tool**	The number of times prescribers engage with the MORE decision support tool in the intervention practices (how many times prescribers choose to look at possible interventions)	Every month during the 6-month pilot phase
	The total number of interventions suggested by the MORE decision support tool in the intervention practices (how many instances of unnecessary or inappropriate medicines)	Every month during the 6-month pilot phase
	Interview and questionnaire containing the standardized System Usability Scale and qualitative questions on usability and usefulness	After completion of pilot phase
**Practitioners follow the recommendations**	The total number of times prescribers change medicines in response to the MORE decision support tool in the intervention practices (ie, remove unnecessary or inappropriate medicines)	Every month during the 6-month pilot phase
	The number of changes sustained or reverted to original prescribing methods within 6 months of the intervention in the intervention practices	Six months after completion of pilot phase
	The difference in number of laboratory tests ordered between intervention and control practices in line with suggestions from the MORE decision support tool	Every month during the 6-month pilot phase
**Reduction in inappropriate prescribing and polypharmacy**	The change in the proportion of patients with inappropriately prescribed medicines^a^ before and after the introduction of the decision support tool (adjusted for age and comorbidities) between intervention and control practices. Specifically: 1. The number and proportion of patients with hyperpolypharmacy (more than nine medicines [2]) before and after intervention 2. The number and proportion of patients with polypharmacy (more than four medicines [ 2]) before and after intervention 3. The average number of medicines per patient before and after intervention	At baseline and at end of the 6-month pilot phase
	The percentage of patients receiving appropriate treatment based on the developed clinical rules in each practice between intervention and control practices	At baseline and at end of the 6-month pilot phase
	Initiation rates of inappropriately prescribed medicines based on the developed clinical rules in each practice between intervention and control practices	Every month during the 6-month pilot phase
	Initiation rates of appropriately prescribed medicines according to the developed clinical rules in each practice between intervention and control practices	Every month during the 6-month pilot phase
**Improvement in patient health**	Changed, maintained, or improved health status measured by number of patient visits to general practices and hospital or emergency department admissions between intervention and control practices	From 6 months before study enrolment to 6 months after completion of the pilot phase
	Deaths in the study year between intervention and control practices	From 6 months before study enrolment to 6 months after completion of the pilot phase
	The number of related referrals, emergency department visits or hospitalizations within 6 months of the intervention in the intervention practices	During the 6-month pilot phase and for 6 months after completion of the pilot phase

^a^This assumes that on consideration of the MORE decision support tool guidance, only inappropriately prescribed medicines will be stopped (therefore, a decrease in inappropriate polypharmacy will occur).

### Cost Analysis

Potential cost savings through analysis of reduced hospitalizations, emergency department visits, and visits to the general practitioner following implementation of the decision support tool will be calculated after 6 months. The costs associated with stopping or starting medicines as recommended by the MORE decision support tool during the study period will also be evaluated. This will be balanced against any consultation slow down’s due to using the tool. There will also be an estimation of the time saved using the MORE decision support tool compared with alternative methods of medicine review.

### Interviews

The intervention group will be interviewed to elicit feedback on the usefulness of the decision support tool and its usability and presentation. This will provide the basis for future improvements for the decision support tool. During the interview a questionnaire will be completed containing the system usability scale, a validated tool for measuring a systems usability [19], to elicit participants’ satisfaction with the decision support tool. Reasons for and against following the decision support tool’s interventions and the differences in managing patients with multiple medicines before and during the use of the MORE decision support tool will also be explored. This will determine whether the decision support tool improves identification of patients with polypharmacy and those who have inappropriately prescribed medicines.

The control group will also be interviewed to understand how they regularly identify and manage patients with polypharmacy. Also, to see who has inappropriately prescribed medicines, what percentage of their patients they estimate belong to these groups, and how often they would undertake a medicine review in an average week.

## Results

Phase one has now been completed and the decision support tool is under development. See Textbox 1 for the clinical rules to be built into the decision support tool. Phases two and three are expected to be finalized early 2016 with implementation and analysis of the decision support tool by October 2016. Results will be reported in December 2016.

## Discussion

### Reducing Inappropriate Polypharmacy

New Zealand general practice is facing increasing challenges in caring for a growing number of patients with long-term conditions. This increases the complexity of primary care interactions and makes high quality medicines review more difficult to achieve within available consultation time frames. It is well recognized that the quality use of medicines leads to decreased medicine interactions, reduced health care use (including hospitalizations), and improved quality of life [72,73]. Therefore, the individualized approach to medicine review undertaken in this study will promote a safe and effective means of practicing.

The premise of optimizing medicines and the reduction of inappropriate polypharmacy is about finding the best available medication at the right dosage and for the shortest possible duration on a case-by-case basis. This decision support tool will assist prescribers in achieving these goals by collating relevant patient information automatically using the prescribing patient management system and making individual recommendations for these complex patients. This will reduce the need for manual processing, the gold standard for reviewing medicines, which is a costly and timely enterprise.

The project partnership with BPAC Inc will allow the transfer of this study from evidence to practice as, if successful, the electronic decision support decision support tool could be directly rolled out to most general practices in New Zealand, through existing BPAC systems and networks. If the MORE module is successful in achieving its goals, the clinical rules could be applied across primary care settings, internationally.

### Study Strengths and Limitations

Using a full range of clinical rules rather than focusing on one condition or drug class is a strength of this study. It allows for inclusion of rules applicable to the whole population and for a wide variety of therapeutic interventions. It also enables further development of the tool without restriction to a singular therapeutic condition. Using real-world locations ensures clinical relevance and applicability of the tool.

This is a fully automated and prepopulated tool. There is no manual data entry required by general practitioners and the patient management system will be one they are currently using in their practice. This will ensure minimal time for setup and the practitioners’ time can be focused on the clinical interventions rather than the technical aspect of inputting data. Moreover, training of practitioners will not need to be extensive and they will already have working knowledge of how this type of alert works.

An extra strength and uniqueness of the study is the content of the tool. It will provide suggested actions for prescribers rather than just highlighting a potentially inappropriate medicine. It is believed this will improve the use of the clinical rules by giving prescribers evidence-based guidance on how these rules should be applied to their patients. The tool will also contain links to patient information and options for printing patient information sheets.

One limitation is the inability to blind the clinicians to the intervention. However, because the analysis of the data will be blinded, objective assessment of results will be possible and is an additional strength.

A further limitation of the study is that the tool is being designed for use with a specific PMS in New Zealand. However, our patient object model allows integration with any PMS API, allowing for the solution to be rolled out internationally. If international roll out is an outcome of this pilot study, further usability studies will be necessary for users of different systems.

The measures of effectiveness are not able to capture the reasons why an alert may be dismissed by a practitioner at the time of consultation. It is hoped that the main reasons alerts are being ignored will be captured through feedback and the user survey. The initial results will help shape the questionnaire and interview of the practitioners so these issues can be explored.

It will be difficult to specify patient deaths and hospital admissions that are not related to the intervention. However, we can investigate the causes of deaths and admission to hospital for those with a medicine-related diagnosis for the purposes of the study.

There is a risk of alert fatigue, particularly if a patient with extensive polypharmacy is triggering many alerts. We have tried to minimize this by ensuring alerts are specific to patients’ characteristics. Furthermore, we will investigate complaints of this nature highlighted in the user feedback reports and work to reduce or eliminate them.

### Conclusions

Optimizing medicines use is not necessarily about reducing the number of medicines below an agreed threshold, but about finding the best available medication at the right dosage and for the shortest possible duration on a case-by-case basis. Measuring reductions in the number of patients experiencing polypharmacy and the average number of medicines per patient will indicate removal of unnecessary medicines and the ‘inappropriate polypharmacy’ from their regimen. This study will establish whether the MORE decision support tool stands up to real world conditions and promotes changes in prescribing practice.

**Clinical Rules to be Built Into the Decision Support Tool.**

**Duplicate therapy** [8]

Stop if duplication of drug class or therapy:

- H_2_-receptor antagonists with proton pump inhibitors
- Duplication of benzodiazepines
- Duplication of antipsychotics
- Duplication of selective serotonin receptor antagonists

**Gastroprotectants** [7,8,20,21]

- Stop proton pump inhibitors if they were prescribed for gastroprotection with nonsteroidal anti-inflammatory drugs, aspirin, or corticosteroid therapy, which has now been stopped
- Consider stopping or reducing dose of proton pump inhibitors prescribed for uncomplicated peptic ulcer disease or erosive peptic esophagitis for >8 weeks

**Chronic constipation** [8,9,22]

In patients with chronic constipation, stop drugs likely to cause constipation if nonconstipating alternatives are appropriate

**Antiplatelets and anticoagulants** [8,9,23-28]

Stop vitamin K antagonist, direct thrombin inhibitor, or factor Xa inhibitors:

- if prescribed for first deep venous thrombosis without continuing provoking risk factors for >6 months
- if prescribed for first pulmonary embolus without continuing provoking risk factors for >12 months

Stop aspirin if taken for primary prevention of cardiovascular disease if risk is <20% and there is no personal history of cardiovascular disease (ie, angina, myocardial infarction, percutaneous coronary intervention, coronary artery bypass grafting, transient ischemic attack, ischemic stroke, or peripheral vascular disease)

**Medicines in the elderly** [8,9,29-37]

- Stop long-term treatment with loop diuretics treating gravitational edema (unrelated to congestive heart failure) in the elderly. If pharmacological treatment necessary, prescribe as required
- Reduce spironolactone if >25-mg daily in elderly patients with congestive heart failure or with creatinine clearance less than 30 mL/min
- Review patients >85 years taking statins for >5 years for primary cardiovascular prevention
- Stop benzodiazepines or zopiclone if taken for treatment of insomnia, agitation, or delirium in adults aged >65 years
- Stop antipsychotics for behavioral problems of dementia unless nonpharmacological options have failed, and patient is a threat to themselves or others
- Stop first-generation antihistamines in patients >75 years
- Stop orphenadrine in patients >75 years

**Antipsychotics, antidepressants, and hypnotics **[8,9,27,31,38-40]

- Stop all antipsychotics (except for quetiapine and clozapine), metoclopramide, prochlorperazine, and promethazine in patients with Parkinson's disease
- Reduce citalopram doses ˃40-mg daily
- Stop benzodiazepines or zopiclone if taken for ≥4 weeks (unless for seizure disorders, rapid eye movement sleep disorders, benzodiazepine withdrawal, alcohol withdrawal, severe generalized anxiety disorder, periprocedural anesthesia, end-of-life care)

**Analgesics** [31,41-47]

- Review opioids if being prescribed long term (>3 months) for nonmalignant pain
- Stop two different types of long-acting opioid
- Stop codeine or tramadol if prescribed with a strong opioid
- Stop combination paracetamol and codeine products and prescribe individual components based on the World Health Organization pain ladder

**Nonsteroidal anti-inflammatory drugs** [8,9,27,48,49]

- Review stopping nonsteroidal anti-inflammatory drugs:
  - if used for ˃3 months in patients over 75 years
  - if used for ˃3 months in patients for symptom relief of osteoarthritis pain where paracetamol has not been tried
- Stop nonsteroidal anti-inflammatory drugs:
  - if prescribed with concurrent antiplatelet or prescribe proton pump inhibitor prophylaxis
  - in patients with heart failure
  - in patients being treated with a diuretic or an Angiotensin Converting Enzyme inhibitor or Angiotensin Receptor Blocker
  - if patients already taking a nonsteroidal anti-inflammatory drug (ie, duplicate therapy)

**Gout **[8,50-52]

- Prescribe allopurinol for patients with a history of recurrent episodes of gout (recurrent nonsteroidal anti-inflammatory drug or colchicine prescriptions), or a suitable alternative if allopurinol contraindicated
- Alert if patient is on allopurinol and no uric acid levels or renal function tests for >1 year
- Maximize dose of allopurinol or add additional therapy if uric acid not ˂0.36 mmol/L

**Bisphosphonates** [8,53,54]

- Consider stopping bisphosphonate treatment or a ‘drug holiday’ after continuous use for >5 years (for treatment or prevention of osteoporosis) if bone mineral density stabilized
- Consider initiating bisphosphonates in patients taking long-term systemic corticosteroid therapy

**Metformin** [31,55]

Adjust dose of metformin in renal impairment

**Seasonal influenza vaccine** [8,56-59]

Recommend an annual seasonal influenza vaccine to:

- patients ≥65 years of age
- patients with ischemic heart disease
- patients with congestive heart failure
- patients with rheumatic heart disease
- patients with Transient Ischemic Attack/Stroke
- patients with asthma, if on a regular preventative therapy
- patients with chronic obstructive pulmonary disorder
- patients with diabetes
- patients with any cancer, excluding basal and squamous skin cancers if not invasive
- patients with human immune deficiency virus
- transplant recipients
- pre and post splenectomy patients
- pregnant patients

**Interactions** [8,27,31,60-64]

Alert for the following interactions:

- Tricyclic antidepressants and selective serotonin receptor blockers
- Beta-blocker with verapamil or diltiazem
- Concomitant use of two or more antimuscarinics
- Concomitant drugs that prolong the QT-interval

**Monitoring** [8,65-71]

- Monitor potassium levels if not done >6 months in patients taking spironolactone with potassium sparing drugs
- Stop potassium supplements if serum potassium >4.0 mmol/L and if cause of hypokalemia resolved. Consider follow up potassium levels after cessation
- Alert if on lithium and no thyroid function tests, renal function tests, serum lithium levels, or sodium levels for >6 months, or no calcium levels or electrocardiogram undertaken for >1 year
- Alert if on an Angiotensin Converting Enzyme inhibitor and no renal function or serum potassium for >1 year
- Alert if on an atypical antipsychotic for schizophrenia and no CV assessment, full blood count, urea and electrolytes, liver function tests, lipid profile, weight measurement, fasting blood glucose, or prolactin levels for >1 year

## Appendix

Multimedia Appendix 1 Grant agency peer-review reports.

## Abbreviations

API: application program interface
BPAC: Best Practice Advocacy Centre
EHR: electronic health record
MORE: medicines optimization/review and evaluation
PMS: patient management system
SNOMED CT: systematized nomenclature of medicine clinical terminology
