# Supplementary material for: Using an Electronic Decision Support Tool to Reduce Inappropriate Polypharmacy and Optimize Medicines: Rationale and Methods
Source: JMIR Res Protoc. 2016 Jun 10;5(2):e105. doi: 10.2196/resprot.5543 (PMC4920961; doi:10.2196/resprot.5543)
Supplement: Multimedia Appendix 1 [file resprot_v5i2e105_app1.pdf]

UNIVERSITY of OTAGO
Te Whare Wānanga o Otāgo
NEW ZEALAND

18 January 2016

Dear Editor JMIR Protocols

Re: Grant agency peer-review reports

Please see below for two rounds of peer-review from New Zealand's largest grant funding agency, the Health Research Council (http://www.hrc.govt.nz/). Also attached are the primary investigator's rebuttal and letters answering the reviews. These grant applications undergo significant review from experts in the field from both New Zealand and Australia.

You will see that this project was funded by the HRC, under a health partnership grant scheme.

Sincerely,

Dr Alesha Smith
School of Pharmacy

School of Pharmacy
Te Kura Matauraka Wai-Whakaora
University of Otago
PO Box 56, Dunedin 9054, New Zealand
Tel +64 3 479 5052 · Email alesha.smith@otago.ac.nz

Health Research Council of New Zealand

Referee Report - Applicant version

Assessing Committee: .2014 RPNZHD HRC Ref# 14/730

Referee#: 10

PROPOSAL DETAILS:
14/730 Dr Alesha Smith
Integrating patient data to optimise medicines and reduce polypharmacy
(University of Otago)

SCIENTIFIC MERIT

This research is highly innovative. It aims to develop a real-time patient-centred tool for prescribers to improve use of medicines within the health-care encounter. Medication misadventure in the majority of developed countries contributes more to health burden than common diseases such as heart failure, diabetes, or asthma, thus the research has significant potential to improve health and reduce harm. If the research is successful, this work will be significant internationally, because of the novelty of real-time patient-centred approach.

DESIGN AND METHODS

The project plans a phased approach, using literature review and clinical consultation to develop the algorithms to be implemented. The research team work with an established partner organisation experienced in developing clinical software to develop the tool. Face and content validity are established using historical data to ensure feasibility, with the testing phase being undertaken in two general practices across the country and assessed against a comparison group of two practices. The study would be strengthened with more control practices. The study is appropriately powered for changes in poly-pharmacy, the endpoint intended to be assessed. The study is not powered for assessing changes in health outcomes, this should not be seen as a limitation as the study was not designed for this. If the study is successful, larger studies that assess the impact on health outcomes would be required.

TEAM CAPABILITY - RESEARCH OUTCOMES

The lead investigator is an early career researcher, with a significant track record relative to opportunity spanning Australia, the UK and New Zealand. The research team is an impressive mix of clinicians and researchers spanning the disciplines of pharmacy, pharmacoepidemiology, general practice, biostatistics, and clinical pharmacology and is in partnership with BPAC, an organisation with significant track record in the area. The more experienced researchers should be able to provide appropriate mentorship for Dr Smith and the team's track record demonstrates their capacity to manage grants of this nature.

TEAM CAPABILITY - RESEARCH UPTAKE

If this research is successful, it will be highly usable. The planned phased method maximises the chance of development of a successful product and the significant number of researchers from a general practice background will ensure the likelihood of a tool with appropriate application to general practice

IMPACT ON NEW ZEALAND HEALTH DELIVERY

If the tool is successful it will be able to be rolled out to general practices across New Zealand where it is likely to have a significant impact quickly. Medication use is the most common intervention in health care and consequent with that medication error and adverse drug events are most common cause of harm. Reducing errors and inappropriate prescribing will contribute to reduced harm, improved outcomes and reduced costs.

OVERALL/GENERAL COMMENTS (To Applicant, HRC and Committee)

I think overall this is an impressive grant. The project is well thought through and the research team and partner organisation appropriate to the task. The only limitation of the research is that phase four is a small study (4 practices) which means health outcomes cannot be assessed. This arises solely due to the maximum funding limit as the inclusion of more practices would add considerably to costs.

Printed at:..2014-06-24 20:27:36 - ref 11637

PROPOSAL DETAILS:
14/730 Dr Alesha Smith
Integrating patient data to optimise medicines and reduce polypharmacy
(University of Otago)

SCIENTIFIC MERIT

The extent of inappropriate medicine use, especially in those taking multiple medicines represents a significant health challenge for all health care professionals involved, patients and carers and puts a significant burden on health care systems. Hence interventions designed to address this are of high scientific, professional and practical importance. Hence the importance of this proposal. If successful, this proposal has the potential to significantly advance practice. The first two objectives of this study involve significant development and the third objective describes a feasibility study. Notwithstanding the feasibility study the investigators have included a null hypothesis with sample size calculation in this proposal. The approach is original and builds on the existing work of bpac.

DESIGN AND METHODS

The proposal describes a justification for the different aspects of the research design and methods. The research team and collaborators is more than capable of conducting this project. However there is a significant risk for this proposal in that no or limited preliminary data has been presented and that the successful implementation of the proposal includes all aspects of the development, evaluation and assessment of the EDS module. Specifically the project design comprises four phases which are conducted in sequence. Delay or deficiencies in any of the phases will therefore have an impact on subsequent phases of the project. Also, presumably some aspects of the project, such as the conduct of literature reviews and compilation of prescribing resources, should/could have already been undertaken.
The proposal differentiates between polypharmacy and hyperpolypharmacy. A justification for the use of a higher cutoff of 10 medicines per patient has not been detailed. The use of a hyperpolypharmacy cutoff may present challenges in the identification of suitable patients to meet the sample size within the two general practice intervention sites, given an estimated 11% rate of hyperpolypharmacy.
The reference to support the influence of EDS on prescribing is the bpac website. Could an alternative or additional peer reviewed reference have been used?
The proposal provides little detail of how or what specific individual data will be used in the MORE module to the extent to which this data will allow for a meaningful individualized recommendation versus a “generic” recommendation.
Multimorbidity is mentioned throughout the proposal including the aim of this study, however how multimorbidity will be considered in the MORE module has not been detailed. The example clinical rules listed in the proposal seem to have an individual medicine or drug class as the unit of analysis and do not discuss conditions or the handling of multiple conditions. Will the MORE module consider factors such as patient preference or a shared decision making approach?
The proposal implies that the MORE module will minimize the need for a manual review of medicines and

be time saving for GPs. Could it be the case that presenting medicine recommendations to GPs in complex (hperpolypharmacy) patients with multimorbidity, may require more GP time to consider the recommendations? (given that GPs are unlikely to blindly implement the recommendations made by the module).
The MAI does not identify specific medicines that are often prescribed inappropriately. Unlike Beers criteria and STOPP/START it is an implicit tool rather than an explicit tool.
The second part of objective 1 is unclear.
Is this a feasibility or pilot study?
Although paediatric and adolescent patients may be selected, the hyperpolypharmacy criterion may rule out many who may benefit from EDS due to complexity in the use of medicines.
Regarding the (boxed) example of the MORE module – would the recommendation regarding the use of omeprazole in a child come up if the PPI was being prescribed for the first time? Based on the prompt provided it should. What would happen for the same patient if they did not have (GI) symptoms or ADRs and they were currently taking the PPI? Would the alert only come up if the patient was taking 10 or more medicines?

TEAM CAPABILITY - RESEARCH OUTCOMES

The research team combines an excellent balance of academics, practitioners, and decision support/IT implementation. The combined skill set of this group is more than capable of effectively conducting this proposal. It is noteworthy that the PI, an early career researcher, has an experienced academic mentor to support her in all aspects of the research.

TEAM CAPABILITY - RESEARCH UPTAKE

The publication track record of the research team is significant and spans the related areas of pharmacy, clinical pharmacology and general practice, all of which are of direct relevance to this proposal. The professional standing of the research team is well established and evident through numerous current or previous leadership positions of key organisations and committees at both the local and national levels. There is evidence that some members of the research team have worked together previously, in the form of co-authored publications. More broadly the core research team will be supported by a list of national and international collaborators. It is noted that most of this support will be offered in-kind. Notwithstanding the significant records of these external collaborators, the proposal does not articulate in detail how the core research team will practically facilitate the involvement of the external collaborators or the expected time commitment of them. Furthermore, given that the proposal has been described as a feasibility study, it may be argued that additional local NZ experts may arguably be in a better position to provide input into content and feasibility issues. Nevertheless, if successful this proposal, as the investigators note, has the potential to be rolled out due to the collaboration with bpac. However, even if this proposal was successful, it would seem prudent to repeat and upscale the project before national implementation.

IMPACT ON NEW ZEALAND HEALTH DELIVERY

The extent of inappropriate medicine use, especially in those taking multiple medicines represents a significant health challenge for all health care professionals involved, patients and carers and puts a significant burden on health care systems. Hence interventions designed to address this are of high scientific, professional and practical importance. Hence the importance of this proposal. If successful, this proposal has the potential to significantly advance practice.

OVERALL/GENERAL COMMENTS (To Applicant, HRC and Committee)

No additional comments or questions.

Printed at:..2014-06-25 17:51:35 - ref 11637

Health Research Council of New Zealand

Referee Report - Applicant version

Assessing Committee: .2014 RPNZHD HRC Ref# 14/730

Referee#: 33

PROPOSAL DETAILS:
14/730 Dr Alesha Smith
Integrating patient data to optimise medicines and reduce polypharmacy
(University of Otago)

SCIENTIFIC MERIT

I was excited to read this application which deals with an important health issue, and is proposing a pragmatic response.

In relation to scientific merit the primary outcome is vague and appears to comprise four (five if you count differences in both group 1 &2) quantitative co-primary outcomes and one qualitative outcome. Although the mixed methods approach is potentially valuable, usually a single pre-specified primary outcome is preferred (or, at most, a co-primary outcome).

DESIGN AND METHODS

I was concerned about the design of the study. Although the BPAC team sound excellent, I was concerned that Phases II and III do not explicitly include testing by GPs. Presumably GPs should be involved in the development of the module. You could prejudice success in Ph IV if GPs are not adequately involved in Phase II (you acknowledge all of the limitations in the literature regarding alerts being overridden by busy Drs – I thus feel it's incumbent on the team to pilot test the module with GPs in Phase II, to determine that GPs feel it meets their needs).

I was likewise not at all convinced of the rational for limiting validation to the technical aspects (ie validation using fictitious patients, as opposed to determining acceptability etc by GPs piloting the tool).

I was also concerned about the statistical methods. Although intervention sites will be randomly selected, they will then be matched with other practices. So the design is quasi-experimental. The power calculation does not account for clustering at practice level, which would appear to be relevant. I note that a statistician is involved but I feel these points need to be explicitly addressed in the rebuttal as I do not have confidence in the analytic approach as presented currently.

It's not clear why the proposal target hyperpolypharmacy (rather than polypharmacy), and will only comprise 10 clinical rules (these aspects seem arbitrary – what if the 11th clinical rule appears to be important?).

TEAM CAPABILITY - RESEARCH OUTCOMES

The team appears strong and capable with a good mix of GP, pharmacy, pharmacology, technical and statistics input. I was surprised that leading NZ figures in the field of de-prescribing (eg Mangin has published with Garfinkel) were not even listed in the collaboration section.

TEAM CAPABILITY - RESEARCH UPTAKE

I was initially uncertain (thinking BPAC was a commercial provider) but after reading the BPAC website was convinced of this aspect of the application. Thus, although there is little actual concrete detail in the application, BPAC appears to be able to engage end users successfully.

IMPACT ON NEW ZEALAND HEALTH DELIVERY

Potential impact is limited by the scientific and design concerns listed. If the intervention is not shown to be successful it could be because the development was not rigorous, or because the efficacy study was poorly designed or analysed. Otherwise the application is strong in terms of the partnership with BPAC and potential for impact through wide uptake of a successful module.

OVERALL/GENERAL COMMENTS (To Applicant, HRC and Committee)

nil additional

Printed at:..2014-06-20 21:05:11 - ref 11637

Health Research Council of New Zealand

Referee Report - Applicant version

Assessing Committee: .2014 RPNZHD HRC Ref# 14/730

Referee#: 67

PROPOSAL DETAILS:
14/730 Dr Alesha Smith
Integrating patient data to optimise medicines and reduce polypharmacy
(University of Otago)

SCIENTIFIC MERIT

This project targets the problem of polypharmacy and reducing the risk of adverse drug reactions. It is difficult to assess how effective the intervention is likely to be given that the rules to be included in the MORE module have not been defined. Some initial work on the rule development would have greatly strengthened the application and assisted reviewers in assessing the likely impact of the intervention. It would be important to establish that interventions in general practice based on these rules will result in reduced prescribing in people with hyper-polypharmacy. This was not clear in the application.

DESIGN AND METHODS

I felt that the timeline for this application was not feasible. For example, the intervention is to run for 6 months until Sept 2015, with patients being followed for up to 6 months to determine outcomes. It is not clear to me from the timeline that this would be possible given the project ends in March 2016. Given the rules have not been established, it is not clear how many patients the investigators will need to follow up. Conceivably, with 10 rules, this could included a large number of patients. Will the budget be sufficient for these follow up calls given the time involved? How many patients do the investigators expect to follow up?

TEAM CAPABILITY - RESEARCH OUTCOMES

Collectively, the team has the experience necessary to conduct the proposed research. However, I question whether the project can be completed within the suggested timeframe.

TEAM CAPABILITY - RESEARCH UPTAKE

The application demonstrates meaningful engagement with end users and is likely to result in good participation.

IMPACT ON NEW ZEALAND HEALTH DELIVERY

This is not clear from the application. As previously stated, this would depend on the rules chosen, and a number of other factors, including uptake by GPs, potential to reduce hyper-polypharmacy, and the degree of patient benefit derived from application of the rule. I feel the investigators need to provide more evidence on each of these links for their chosen rules before this is implemented/funded.

OVERALL/GENERAL COMMENTS (To Applicant, HRC and Committee)

This application would be greatly strengthened by more clarity on the rules to be chosen. This phase was a part of the project, but the lack of clarity in the application makes it difficult to assess the feasibility of the project. The timeline, especially for follow-up of patients is also a major concern.

Printed at:..2014-06-23 14:48:53 - ref 11637

DO NOT DELETE OR MODIFY THIS TABLE!			
**Applicant**	Dr Alesha Smith	**Respond by:**	---
**Assessing Committee**	2014 RPNZHD	**HRC Reference**	14/730
**Title of Research**	Integrating patient data to optimise medicines and reduce polypharmacy		

We would like to thank the referees for their positive comments including:

1. **The research team**- suitability, capability and experience of the team spanning multiple disciplines (referee# 67, 10, 33 & 27)
2. **The impact on New Zealand health delivery and international significance** of the proposed project which will contribute to reducing harm and improving outcomes and reducing costs (#10)
3. **The strength of the partnership between bpac^{nz} and the University of Otago** bringing together experts in IT, clinicians and researchers (#27, 10, 33)
4. **The pragmatic design of the project** and that the developed tool would be highly useable (#10) with wide uptake (#33) and meaningful engagement with end users (#67)

The referees had some comments and questions around the following topics:

1. Referees # 67 and #33 requested further information around phase 1 - the development of the **clinical rules** in the MORE module. Limited information was given here due to space constraints. From our initial work, we anticipate that 10 rules will be chosen using literature searches, expert clinical advice and internationally validated prescribing quality tools such as STOPP/START to enhance the quality use of medicines (#27). This phase has not been completed as funding is needed to for time and expertise to evaluate the literature and validated tools. If it is deemed that a rule will benefit a large target group, then the recommendation will appear even if the patient does not have hyper-polypharmacy (e.g. proton pump inhibitors (PPIS) in children) as our intention is to improve quality use of medicines overall (#27).

2. **Individualised data** used to make recommendations (#27). Individual patient data such as age, sex, co-morbidities, current and previously prescribed medicines, test results and other factors such as CVD risk assessment and renal impairment will automatically be included by the MORE module. This will allow an individualised recommendation to be made to the prescriber (e.g. this patient has reduced renal function - consider reducing the dose to xx or changing this medicine to xx) rather than a generic, irrelevant or broad recommendation (e.g. prescribe with caution in renal impairment) that is likely to be ignored.

3. **Timelines** were questioned by referees #67 and #27. The timelines are compliant with the full 18 months available for the research, with phases adhering to strict timelines. BPAC and the researchers are used to complying with tight deadlines and have proven this with the successful completion of other grants and commercial projects. Some phases of the project are already underway e.g. ethics applications and Maori consultation, which form the first two months of the project timeline, to allow some contingency to the proposed method.

4. **Sample size** #67 and #33. It is very difficult at this stage to undertake a power calculation that accounts for clustering (#33) as we will not know the patient size of each cluster until after recruitment; therefore, we cannot determine the intra-cluster correlation coefficient. Referee #10 agreed that we have adequately powered the study to measure changes in polypharmacy. Although this is a small sample size of 4 practices (2 intervention, 2 control), we believe that patient cohort of between 20,000-40,000 individuals in total will provide enough information needed to determine a sample size for a large nation-wide randomised control trial, if the proposed study is successful. A small sample will also allow us to gain in-depth knowledge from participating prescribers around the usability of the tool, the barriers, facilitators and future recommendations.

5. **Experiment design and outcome measures**. This study is not quasi-experimental as suggested by #33. The intervention practices will be randomly assigned, then matched to control practices. The outcome measures for this study are robust quantitative data that address

**DO NOT DELETE OR MODIFY THIS TABLE!**			
**Applicant**	Dr Alesha Smith	**Respond by:**	---
**Assessing Committee**	2014 RPNZHD	**HRC Reference**	14/730
**Title of Research**	Integrating patient data to optimise medicines and reduce polypharmacy		

the primary outcome of determining the usability of the MORE module. We consider the design and outcome measures appropriate for this stage of developing an innovative and novel tool.

6. **Polypharmacy** vs **hyper-polypharmacy** (#27 and #33). The ultimate outcome from this project is to improve the quality use of medicines in New Zealand. Improvement measures may include reduction in the number of patients with hyper-polypharmacy (10 or more concurrent) medicines and/or polypharmacy (5 or more concurrent medicines). We specifically chose hyper-polypharmacy as the primary outcome measure for this study as these patients are most likely to benefit from a medicines review, yet often most difficult to review due to the high number of medicines. Hyper-polypharmacy patients are at increased risk of hospitalisation due to drug-disease, drug-drug or drug-food interactions. ≥5 medicines would capture a huge number of patients, many who will be taking 5-9 medicines considered essential for their condition/s e.g. usually ≥5 medicines are required as standard treatment post heart attack.

7. **Multimorbidity** was raised by referee #27. Multimorbidity is not a measured outcome in this study but it contributes indirectly. Multimorbidity (multiple diseases) is often considered a consequence of aging, and is broadly thought to be a cause of polypharmacy due to improved treatment options for diseases.

8. The MORE module will not account for **patient preferences and shared care** (referee #27), but it will allow prescribers more time in their consultations to consider these factors and will prompt prescribers to do this. The MORE module will automatically review the patient's medicines, taking this time consuming task away from the prescriber and freeing up more time for discussion with the patient.

9. **Further reference** (#27). Electronic decision support development in New Zealand is relatively new, but an HRC grant to measure health outcomes and changed general practice behaviour regarding TIA/stroke diagnosis and management showed that EDS use resulted in fewer deaths and decreased health costs. Ranta A, Dovey S, Weatherall M, O'Dea D, Gommans J, Tilyard M. Efficacy and Safety of an Electronic Decision Support Tool for Diagnosis and Management of TIA and Minor Stroke: A Cluster Randomised Controlled Trial in General Practice. *Br Med J* [submitted May 9, 2014].

10. Referee #27 commented on the strong **connections and international collaborations** with leading experts for this project. Referee #33 commented about the lack of national collaborators, however all members of the project team currently are leaders in this field and also work with and have established networks with national researchers in primary care and quality use of medicines; in particular, Dr Tordoff collaborates with D Mangin (as mentioned by referee #33). This national expertise can be accessed as needed. It is expected that those in the clinical advisory group would participate in 2 x ½ day meetings during the study project and an additional ½ day, accessed as required to review documents or provide feedback (#27).

11. Referee #33 asked about the **involvement of GPs in the development of the tool**. Dr Lloyd, a practicing GP, will oversee the development of the MORE module as the divisional head of information services at BPAC. Prof Tilyard, a practising GP, will also assist with the development. As outline in phase 3, the MORE module will be validated using a historic database; GPs will also be invited to pilot and comment on the module.

We would like to thank the referees for their overall comments that this is an impressive grant (#10) which has the potential to impact NZ health delivery through wide uptake and participation (#33, #67) and to significantly advance practice (27).

hrc

28 August 2014

Dr Alesha Smith
Pharmacy
Health Sciences
University of Otago
Adams Building
Fredderick St
Dunedin 9016

Dear Alesha

14/730 Integrating patient data to optimise medicines and reduce polypharmacy

The Research Partnerships for New Zealand Health Delivery (RPNZHD) Assessing Committee has now completed its assessment of all full applications received in response to the 2014 Request for Proposals (RFP) for this funding initiative. I am pleased to inform you that you have been selected as a preferred provider for this research contract, contingent upon a satisfactory response to the feedback outlined below.

All applications were reviewed during a recent assessment meeting, taking into consideration peer review reports and applicant rebuttal. The Assessing Committee wish to acknowledge the time and effort that went into the development of your proposal.

General comments and questions from the Assessing Committee are listed below. Please note the Health Research Council of New Zealand (HRC) will require a satisfactory response to the questions prior to finalisation of a contract:

- This application addressed an important area for New Zealand health delivery and the committee acknowledged that targeting this issue at a general practice level would be very useful.
- The committee commented that this was a strong research team, with a good track record, and noted there were good links for successful dissemination through BPAC.
- The committee would have liked to have seen more detail on how GPs will manage digesting the recommendations about polypharmacy and then discussing them with their patients within a 15 minute consultation and would have liked a discussion around the impact of this on improving quality service delivery in primary care.
- The committee was uncertain of the key focus of the programme – the management of hyper-polypharmacy; polypharmacy; drug reactions, or inappropriate prescribing?

- The committee noted that the proposal plans to identify 10 clinical rules in the MORE module. The committee requested that the applicants provide clearer specification of these rules and the distinct end-points of the research. For example, reduced hyper-polypharmacy appears to be a measure of the first proposed rule, but not of the other nine.

The Committee will await your written response to the above issues before making the final decision on funding. Please provide your response within three weeks of the receipt of this letter. If you do not wish to proceed further in the assessment process, please inform the HRC immediately.

Your response should be emailed to Luke Garland, Project Manager, Research Partnerships, at the HRC. If you wish to clarify any points raised in this letter, please contact Luke Garland on (09) 303 5214 or email lgarland@hrc.govt.nz. The Assessing Committee regards all correspondence pertaining to this initiative as confidential until the final decision on the research provider has been reached.

Yours sincerely

Lex Davidson
Acting Chief Executive

cc Dr Gavin Clark, Director Research and Enterprise, University of Otago

g:\corres\chief executive\2014\correspondence\research partnerships\0828 smith.docx

UNIVERSITY of OTAGO
Te Whare Wānanga o Otāgo
NEW ZEALAND

Health Research Council
110 Stanley St
Auckland, 1010

Re: 14/730 Integrating patient data to optimise medicines and reduce polypharmacy

Dear Mr Davidson

Thank you for your letter regarding our application for a Research Partnership for New Zealand Health Delivery. Please find below responses to the questions raised in this letter.

1, 2. Importance and Research team
Thank you for acknowledging that targeting the issue of polypharmacy at a general practice level is a useful approach. Thank you also for recognising the strong research team behind this grant, our positive track record and the good links for successful dissemination though BPAC.

3a. Managing the MODULE recommendations in a 15 minute consultation

The number of available clinical decision support tools has significantly increased over the last 5 years; therefore GPs are very familiar with using and managing these tools within a 15 minute consultation. To-date clinical decision support tools developed by BPAC have been used over two million times during general practice consultations. The proposed research has also incorporated features that will hopefully assist GPs in using the MORE module, including:

- Saving time: The MORE module will automatically review each patient's medicines for the ten clinical rules, taking this time consuming task away from the prescriber and freeing up more time for discussing the information with the patient.
- Education: Intervention practices will also receive written education materials and face-to-face academic detailing on the different clinical rules recommended by the MORE module, meaning less GP time spent digesting the information during the consultation thus more time to implement the changes and consider patient preferences.
- Prescriber resources: We will provide a printed hand-book/guide for GPs outlining the key messages for each of the clinical rules. They can then have this on-hand to refer to during a consultation, if needed.
- Patient information: Links to validated patient information will be available through the MORE module, which prescribers can print for patients or refer them to the listed websites/information. Examples of potential patient resources may include information sheets from *Health Navigator NZ*[1] or a modified version of the *Personal Decision Guide for Medicines* available from the national prescribing service in Australia.[2]
- Evaluation: As part of the qualitative evaluation of the MORE module (through focus groups and/or interviews), we will obtain feedback on how GPs integrated the module into their consultation and its impact on time management.

3b. Impact on improving quality service delivery

School of Pharmacy
Te Kura Matauraka Wai-Whakaora
University of Otago
PO Box 56, Dunedin 9054, New Zealand
Tel +64 3 479 5052 · Email alesha.smith@otago.ac.nz

A recent BPAC/University of Otago study on Transient Ischaemic Attacks funded by HRC showed that the use of clinical decision support tools in New Zealand can save money for the healthcare system and save the lives of patients in primary care. This study demonstrated that a clinical decision support tool allowing GPs to implement evidence-based care rapidly without immediate reliance on specialists could improve key outcomes for patients and reduce the burden of stroke, providing strong evidence for widespread implementation.[3]

4.Key Focus

The key focus of this grant will be 10 areas (clinical rules) for potentially inappropriate prescribing. These are most likely to occur in patients experiencing polypharmacy (5+ medicines) or hyper-polypharmacy (10+ medicines).

To achieve best results, prescribers need simple, targeted advice and prescribing *alternatives* – not just highlighting inappropriate prescribing. The module will therefore use a 'traffic light system' for **all patients experiencing polypharmacy** to alert prescribers to medicines that are potentially inappropriate (i.e., related to a clinical rule), based on each patient's individual data. For example, red indicates strong level of evidence supporting change; orange - the potential for change; and green –no recommendation for change at this time (no related clinical rule).

5a. Clinical Rules

As stated in the grant application, a three-month period will be dedicated to developing the clinical rules for this study. This phase requires funding to provide for time and expertise to evaluate the literature and validated tools in this area. Likely sources of these rules will be bpac^{nz} areas of focus over the last two years e.g. use of PPIs, recent research into potentially inappropriate prescribing in New Zealand and internationally validated tools e.g. STOPP/START.[4-6] Thus it is premature to specify the clinical rules at application stage.

5b. End-Point

Our partner, BPAC, requires the distinct end-point of this study to be the development and testing of the MORE module to a stage where it has the potential to translate evidence into practice throughout New Zealand primary care. Secondary endpoints related to the clinical rules will be determined once the clinical rules have been developed: e.g. secondary endpoints might include a reduction in the use or dose of a particular medicine (depending on the clinical rule).

In Conclusion

BPAC and the University of Otago have built a strong research collaboration and have a successful track record in research relating to quality and safety in primary care. This study will complement the previous and current projects shown in table 1 below, and continue to build research capacity and develop knowledge relevant to the health needs of New Zealanders.

Table 1. Recent BPAC/The University of Otago research projects

Health Research Council	2007	*Utility of primary care computer records for product vigilance*	**Tilyard M, Dovey S,** Tomlin A, **Reith D**	$91,887
World Health Organisation: World Alliance for Patient Safety	2009	*Assessing and improving safety culture in primary care practices*	Wallis K, **Dovey S**	US$15,000
Health Research	2011	*RCT of Efficacy and Safety of*	Ranta A, **Dovey S**,	$624,846

Council		*TIA Electronic Support Tool (FASTEST) Trial*	**Tilyard M**, Cariga P, Fink J, Campbell J	
Health Research Council	2011	*Patient safety in New Zealand general practices: Records review study [Feasibility Grant]*	Dovey S, Wallis K, Buetow S, Williamson M, Cunningham W, Lillis S, **Reith D, Tilyard M**	$149,275
Health Research Council	2014	*Patient harms in New Zealand general practices: Records review study*	**Dovey S**, Wallis K, Williamson M, Cunningham W, Lillis S, **Reith D, Tilyard M**, Eggleton K, McMenamin A	$1,174,690

If you have any questions or need further information, please do not hesitate to contact me.

Sincerely,

Dr Alesha Smith
School of Pharmacy
University of Otago
Dunedin

Phone: 03 479 5052
Email: alesha.smith@otago.ac.nz

References

1. Health Navigator New Zealand. Available from: www.healthnavigator.org.nz/medication/p/proton-pump-inhibitors/ (Accessed Sep, 2014)
2. NPS Medicinewise. Available from: www.nps.org.au/topics/how-to-be-medicinewise/making-wise-choices-about-medicines (Accessed Sep, 2014)
3. Ranta A, Dovey S, Weatherall M, O'Dea D. Efficacy and safety of a TIA/stroke electronic support tool (FASTEST) trial: study protocol. ImplementSci 2012; 7:107.
4. bpac[nz]. Best Practice Journal. Available from http://www.bpac.org.nz/BPJ. (Accessed September, 2014)
5. Nishtala P, Bagge M, Campbell J, Tordoff J. Potentially inappropriate medicines in a cohort of community-dwelling older people in New Zealand. Geriatr Gerontol Int 2014;14(1): 89-93.
6. Gallagher P, Ryan C, Byrne S, Kennedy J, O'Mahony D. STOPP (Screening Tool of Older Person's Prescriptions) and START (Screening Tool to Alert doctors to Right Treatment). Consensus validation. Int J Clin Pharmacol Ther.2008;46(2):72-83
